# Moral Distress, Moral Resilience, and Organizational Factors in Predicting Consideration of Leaving Among Nurses Working in Infectious Isolation Wards: A Logistic and Machine‐Learning Analysis

**DOI:** 10.1155/jonm/7280726

**Published:** 2026-10-29

**Authors:** Shiyi Li, Mianli Li, Ting Du, Lefei Wang, Jiajun Zhou, Qimin Xie, Bixia Huang, Zhihui Zheng

**Affiliations:** ^1^ School of Nursing Fujian University of Traditional Chinese Medicine Fuzhou Fujian China fjtcm.edu.cn; ^2^ Nursing Department Third People’s Hospital of Fujian Province Fuzhou Fujian China

## Abstract

**Aim:**

To identify internally stable ethics‐related predictors for consideration of leaving among nurses working in infectious isolation wards and to characterize internal model performance and uncertainty.

**Method:**

In this exploratory cross‐sectional prediction study, 268 nurses working in infectious isolation wards at a specialized infectious disease hospital and several general hospitals in China completed structured questionnaires between July and December 2023. The outcome was a study‐specific item on moral distress–related consideration of leaving, with responses of never considered leaving, considered leaving but did not leave, or considered leaving and subsequently left; the latter two categories were combined for the primary analysis. Twenty dimension‐level ethics‐related predictors were evaluated using elastic‐net penalized logistic regression, classification and regression tree (CART), and random forest models under repeated nested cross‐validation.

**Results:**

Of 268 nurses, 104 (38.8%) had considered leaving because of moral distress, including 2 who subsequently left. Predictor collinearity was substantial (maximum variance inflation factor = 11.75). Moral adversity coping and moral distress related to harm to patients’ interests were retained in 100% of elastic‐net fits, with negative and positive standardized coefficients, respectively; the same two variables accounted for all CART root splits and had the largest model‐specific random forest permutation importance. Internally validated AUCs were 0.747 (95% CI 0.688–0.805) for penalized logistic regression, 0.740 (0.679–0.799) for CART, and 0.761 (0.702–0.818) for random forest, with no statistically significant pairwise differences (all *p* ≥ 0.158). The leading signals remained stable after excluding the 2 participants who had subsequently left and after adding demographic and work‐related characteristics to an extended penalized model.

**Conclusion:**

Consideration of leaving was most consistently associated with moral distress related to harm to patients’ interests and lower moral adversity coping. These internally stable associations highlight ethical experiences and coping demands that warrant further study in nursing management and retention research. Model discrimination was moderate, with some overestimation at higher predicted probabilities; prospective external validation is warranted.

**Implications for Nursing Management:**

Nursing managers should pay attention to ethical concerns involving patients’ interests and nurses’ ability to cope with moral adversity. These findings may help guide the development and evaluation of ethics support initiatives.

## 1. Introduction

Nursing shortages threaten the stability and quality of healthcare systems worldwide [1]. Since the COVID‐19 pandemic, turnover intention among nurses in infectious disease–related settings has attracted growing attention. Studies of nurses working in isolation wards and other infectious care settings have documented substantial workforce and workflow demands, raising concerns about retention and workforce sustainability [2–4]. Because turnover intention is associated with subsequent turnover and signals workforce instability [5], understanding modifiable and nonmodifiable correlates of nurses’ thoughts of leaving remains an important management priority [6].

Turnover intention has been associated with occupational stress, burnout, job satisfaction, and perceived organizational support [7]. Liu et al. identified job satisfaction, satisfaction with pay, conflict with coworkers, and sense of belonging as relevant correlates [8]. Zhang et al. reported higher turnover intention among nurses with burnout and lower turnover intention among those with greater organizational satisfaction and general well‐being; contract employment was also associated with higher odds [9]. Although moral and ethical stressors are increasingly studied in nursing, evidence from infection‐related departments remains limited. This evidence gap highlights the need to examine ethical factors alongside conventional occupational and organizational correlates in settings where ethical demands are especially salient [10].

Nurses working in infectious care settings frequently face restrictive policies, resource‐allocation dilemmas, and tensions between individual care and collective protection [11]. Such situations can cause moral distress when constraints prevent appropriate care, required actions conflict with professional judgment, or personal beliefs conflict with clinical requirements [12]. Moral distress has been associated with turnover intention and consideration of leaving [13, 14], but its sources may differ in psychological salience and predictive relevance. Examining specific dimensions may therefore clarify which ethical challenges are most consistently associated with thoughts of leaving in infection‐related settings.

Previous studies have linked moral distress and turnover intention with emotional exhaustion, value incongruence, and loss of professional meaning [14, 15]. These associations suggest that moral distress should be considered alongside individual and organizational resources. At the individual level, moral resilience is the capacity to maintain integrity and ethical agency during adversity [16, 17]. Moral courage is the willingness to act ethically despite personal or professional risk [18].

At the organizational level, ethical climate and social support shape how nurses interpret and respond to morally troubling situations. A supportive ethical climate promotes value alignment, fair decision‐making, and open communication [19, 20]. Support from colleagues, supervisors, and family members may provide emotional, informational, or practical resources, although its usefulness may depend on how well it matches the ethical and professional challenge [21, 22]. Examining dimensions rather than total scores may help identify the components that contribute most consistently to prediction.

Most studies predicting turnover intention have used regression models that estimate average linear associations. Tree‐based methods can represent nonlinear relationships and interactions without prespecifying their functional form [23]. Penalized regression, classification and regression tree (CART), and random forest were evaluated under a common internal validation framework to compare predictive performance and predictor stability.

This exploratory prediction study examined nurses working in infectious isolation wards in China. It aimed to identify internally stable ethics‐related predictors of moral distress–related consideration of leaving and to quantify uncertainty in model performance and data‐derived patterns.

## 2. Materials and Methods

### 2.1. Study Design and Participants

This exploratory cross‐sectional prediction study used a convenience sample of nurses working in infectious isolation wards between July and December 2023. Participants were recruited from the only specialized infectious disease hospital in Fujian Province, China, as well as several general hospitals. Inclusion criteria were as follows: (1) holding a valid nurse practice license; (2) currently engaged in frontline clinical nursing work in an infectious isolation ward at a participating hospital; and (3) voluntary participation with electronic informed consent. Exclusion criteria were as follows: (1) administrative or managerial nurses not providing frontline clinical care; and (2) nurses temporarily unable to perform clinical duties due to health issues. The original sample size planning used the AUC precision approach of Hanley and McNeil [24], assuming an expected AUC of approximately 0.80, an event rate of 0.35, and a 95% confidence interval half‐width of approximately 0.05, yielding a target of about 230 participants. The completed STROBE checklist for cross‐sectional studies is provided in Supporting File 1 (STROBE‐checklist‐v4‐cross‐sectional).

### 2.2. Measurement

#### 2.2.1. Demographic Characteristics and Outcome Measure

Developed by the research team, the questionnaire included sociodemographic variables (e.g., age, gender, religious belief, marital status, highest education, professional title, night‐shift frequency, years of experience, and monthly income) and ethics training attendance. The study outcome was assessed using one study‐specific item asking whether respondents had ever considered leaving their current post, or had left a previous post, because of moral distress encountered in clinical practice. Response options were “never considered leaving” (*n* = 164), “considered leaving but did not leave” (*n* = 102), and “considered leaving and subsequently left” (*n* = 2). The latter two categories both indicated prior consideration of leaving and were combined for the primary binary outcome.

#### 2.2.2. Chinese Version of the Rushton Moral Resilience Scale (RMRS)

The RMRS, developed by Heinze et al. [25], was adapted into Chinese by Yang et al. [26]. It comprises 16 items across four dimensions: moral adversity coping, relational integrity, moral efficacy, and personal integrity. Each item is rated on a 4‐point Likert scale (1 = *strongly disagree* to 4 = *strongly agree*), with total scores ranging from 16 to 64; higher scores indicate greater moral resilience. The original Chinese version demonstrated acceptable reliability (Cronbach’s *α* = 0.763). In this study, Cronbach’s *α* was 0.775 for the total scale and 0.846, 0.817, 0.817, and 0.452 for moral adversity coping, relational integrity, moral efficacy, and personal integrity, respectively.

#### 2.2.3. Chinese Version of the Moral Distress Scale–Revised (MDS‐R)

Originally developed by Corley et al. [12] and revised by Hamric et al. (2012) [27], the MDS‐R was translated into Chinese by Sun et al. [28]. It includes 22 items across four dimensions: individual responsibility, failure to act in the best interest of patients, value conflict, and harm to patients’ interests. Each item is rated on frequency and intensity (0–4), and scores are calculated as the product of both. Total scores range from 0 to 352, with higher scores indicating greater moral distress. In this study, Cronbach’s *α* was 0.939 for the total scale and 0.905, 0.789, 0.804, and 0.706 for individual responsibility, failure to act in the best interest of patients, value conflict, and harm to patients’ interests, respectively.

#### 2.2.4. Chinese Version of the Nurses’ Moral Courage Scale (NMCS)

Developed by Numminen et al. [29] and translated into Chinese by Wang et al. [30], the NMCS contains 21 items across four dimensions: moral integrity, commitment to providing good care to patients, compassion and genuine presence with patients, and moral responsibility. Items are rated on a 5‐point Likert scale (1 = *not at all true* to 5 = *completely true*), with total scores ranging from 21 to 105. Higher scores indicate greater moral courage. In this study, Cronbach’s *α* was 0.972 for the total scale and 0.906, 0.906, 0.894, and 0.884 for moral integrity, commitment to providing good care to patients, compassion and genuine presence, and moral responsibility, respectively.

#### 2.2.5. Social Support Rating Scale (SSRS)

Developed by Xiao [31], the SSRS assesses three dimensions of social support: objective support, utilization of social support, and subjective support. It consists of 10 items with varying scoring methods depending on item type. Total scores range from 0 to 66, with higher scores indicating stronger perceived social support. In this study, Cronbach’s *α* was 0.830 for the total scale and 0.718, 0.566, and 0.320 for objective support, support utilization, and subjective support, respectively.

#### 2.2.6. Chinese Version of the Hospital Ethical Climate Survey (HECS)

The HECS, developed by Olson [32] and translated by Wang [33], evaluates nurses’ perceptions of hospital ethical climate across five domains: relationships with nurses, patients, managers, the hospital, and physicians. The scale contains 25 items rated on a 5‐point Likert scale (1 = *strongly disagree* to 5 = *strongly agree*). Total scores range from 25 to 125, with higher scores reflecting a more positive ethical climate. In this study, Cronbach’s *α* was 0.976 for the total scale and 0.856, 0.856, 0.923, 0.919, and 0.904 for the nurse–nurse, nurse–patient, nurse–manager, nurse–hospital, and nurse–physician dimensions, respectively.

### 2.3. Data Collection

Data were collected anonymously through the Wenjuanxing online platform. Participants viewed an electronic information and consent form before accessing the survey, and only those who selected “Yes” to indicate consent could proceed. The nursing departments of participating hospitals distributed the survey link through WeChat to eligible nurses. Each IP address was limited to one submission. Hospital coordinators monitored submission rates but could not access individual responses.

### 2.4. Ethical Considerations

Ethical approval was obtained in March 2023 from the Ethical Review Committee of General Hospital of Fuzhou No. 900 Hospital (Approval No. 2023‐048), the Ethics Committee of Fujian Provincial Hospital (Approval No. K2023‐03‐044), and the Ethics Committee of Quanzhou Orthopedic‐Traumatological Hospital (Approval No. IRB‐2023‐047). These were the participating hospitals with independent ethics committees. The remaining participating hospitals did not have independent ethics committees; permission to conduct the survey was obtained from their nursing departments before data collection. All participants provided electronic informed consent before completing the questionnaire. Responses were confidential, personal information was protected, and the data were used only for research.

### 2.5. Data Analysis

#### 2.5.1. Descriptive and Comparative Analysis

Descriptive and bivariate analyses were conducted in SPSS 27.0. Normally distributed continuous variables are presented as mean ± standard deviation and non‐normally distributed variables as median (25th, 75th percentile). Between‐group differences were assessed using independent‐samples *t* tests or Mann–Whitney *U* tests, as appropriate. Categorical variables are reported as frequencies and percentages and were compared using chi‐square tests; ordinal variables were compared using Mann–Whitney *U* tests. All tests were two‐sided.

#### 2.5.2. Predictor Specification and Diagnostic Assessment

The primary outcome was coded 1 for nurses who had considered leaving because of moral distress, including the 2 respondents who subsequently left, and 0 for those who had never considered leaving. The primary predictor set comprised the 20 dimension scores from the MDS‐R, RMRS, NMCS, SSRS, and HECS, reflecting the study’s substantive focus on moral and contextual factors. Total scale scores were excluded because they are linear combinations of their dimensions. Demographic and work‐related characteristics were summarized descriptively and examined in a sensitivity model rather than included in the primary predictor set. Candidate predictors were not filtered according to univariable *p* values. Spearman’s correlations and variance inflation factors (VIFs) were used to describe predictor redundancy. The outcome and all 20 primary candidate predictors were complete; no imputation was required.

#### 2.5.3. Model Development and Internal Validation

Three models were evaluated: elastic‐net penalized logistic regression, CART, and random forest. All models used repeated stratified fivefold outer cross‐validation with five repeats (25 outer validation fits). Hyperparameters were selected within each outer training set using stratified fourfold inner cross‐validation and AUC as the tuning criterion. For the penalized logistic model, continuous predictors were standardized within the training data of each resampling step; the tuning grid included C values of 0.03, 0.10, 0.30, 1, and 3 and elastic‐net mixing parameters of 0, 0.25, 0.50, 0.75, and 1. CART tuning considered maximum depths of 2–4, minimum leaf sizes of 5, 10, or 20, and complexity‐pruning values of 0, 0.005, 0.01, or 0.02. Random forests used 500 trees and tuned the number of candidate features per split (square root, 30%, or 50% of predictors), minimum leaf size (2, 5, or 10), and maximum depth (unrestricted or 4). No class weighting, oversampling, or undersampling was used. A fixed random seed (42) was used for resampling and stochastic estimators to make the analysis reproducible.

#### 2.5.4. Model Performance, Calibration, and Stability Assessment

Each participant’s out‐of‐fold probabilities were averaged across the five outer repeats. Discrimination was summarized using the area under the receiver operating characteristic curve (AUC) and average precision, overall prediction error using the Brier score, and calibration using the calibration intercept, slope, and calibration plots. Subject‐level bootstrap resampling (2000 resamples) provided 95% confidence intervals for performance metrics, and paired subject‐level bootstrap resampling (5000 resamples) was used for AUC comparisons. Accuracy, sensitivity, specificity, and F1 score were averaged across the five outer repeats using thresholds selected by Youden’s index in the corresponding training data. Predictor stability was summarized using elastic‐net selection frequencies and standardized coefficients, CART root split frequencies and thresholds, and test‐fold permutation importance for the random forest. Predictive modeling was performed in Python 3.12.13 using scikit‐learn 1.8.0 and SciPy 1.17.0.

#### 2.5.5. Sensitivity Analyses

Two sensitivity analyses evaluated the robustness of the principal predictive signals. First, the two respondents who had considered leaving and subsequently left were excluded, leaving 266 participants and 102 positive outcomes. Second, an extended elastic‐net model added age, gender, marital status, education, professional title, years of experience, night‐shift frequency, monthly income, and ethics training attendance to the 20 primary candidate predictors. Continuous variables were standardized, and categorical variables were one‐hot encoded within the training data of each resampling step. The repeated nested cross‐validation structure was retained.

## 3. Results

### 3.1. Demographic Characteristics

A total of 268 valid responses were collected. Age data were complete for all participants; ages ranged from 20 to 59 years (mean = 34.49, SD = 8.45). Of the 268 nurses, 104 (38.8%) had considered leaving because of moral distress and 164 (61.2%) had never considered leaving. The 104 positive responses comprised 102 nurses who considered leaving but did not leave and 2 who considered leaving and subsequently left. Additional demographic information is presented in Table 1. No clear demographic or work‐related differences were observed between the two groups in these exploratory comparisons (all *p* > 0.05).

**TABLE 1:** Demographic characteristics of nurses working in infectious isolation wards by consideration of leaving (*n* = 268).

Variable	Total *n* (%)	Considered leaving (*n* = 104) *n* (%)	Never considered leaving (*n* = 164) *n* (%)	*t*/*χ* ^2^/*Z*	*p* value
Age (years)	34.49 ± 8.45	33.76 ± 7.42	34.95 ± 9.04	1.170①	0.243
Gender				0.043②	0.835
Male	12 (4.5)	5 (41.7)	7 (58.3)		
Female	256 (95.5)	99 (38.7)	157 (61.3)		
Religious belief				2.152②	0.142
None	241 (89.9)	90 (37.3)	151 (62.7)		
Yes	27 (10.1)	14 (51.9)	13 (48.1)		
Marital status				2.181②	0.336
Unmarried	89 (33.2)	34 (38.2)	55 (61.8)		
Married	168 (62.7)	68 (40.5)	100 (59.5)		
Divorced/widowed	11 (4.1)	2 (18.2)	9 (81.8)		
Highest education				−0.919③	0.358
Below bachelor’s	146 (54.5)	53 (36.3)	93 (63.7)		
Bachelor’s or above	122 (45.5)	51 (41.8)	71 (58.2)		
Professional title				−1.144③	0.253
Nurse	77 (28.7)	25 (32.5)	52 (67.5)		
Senior nurse	109 (40.7)	45 (41.3)	64 (58.7)		
Nurse‐in‐charge	75 (28.0)	31 (41.3)	44 (58.7)		
Deputy chief nurse or above	7 (2.6)	3 (42.9)	4 (57.1)		
Years of experience				−0.860③	0.390
< 10	101 (37.7)	40 (39.6)	61 (60.4)		
10–20	100 (37.3)	43 (43.0)	57 (57.0)		
≥ 20	67 (25.0)	21 (31.3)	46 (68.7)		
Night shifts/month				−1.652③	0.099
≤ 4	117 (43.7)	37 (31.6)	80 (68.4)		
5–7	76 (28.4)	36 (47.4)	40 (52.6)		
≥ 8	75 (28.0)	31 (41.3)	44 (58.7)		
Monthly income				−0.935③	0.350
< ¥3000	14 (5.2)	7 (50.0)	7 (50.0)		
¥3000–¥5999	47 (17.5)	18 (38.3)	29 (61.7)		
¥6000–¥9000	163 (60.8)	65 (39.9)	98 (60.1)		
> ¥9000	44 (16.4)	14 (31.8)	30 (68.2)		
Ethics training attendance				−1.057③	0.290
Never (0 times/year)	27 (10.1)	9 (33.3)	18 (66.7)		
Occasionally (1 time/year)	125 (46.6)	55 (44.0)	70 (56.0)		
Sometimes (2 times/year)	52 (19.4)	20 (38.5)	32 (61.5)		
Frequently (≥ 4 times/year)	64 (23.9)	20 (31.3)	44 (68.8)		

*Note:* Percentages in the group columns are row percentages. The “considered leaving” group includes 102 nurses who considered leaving but did not leave and 2 who considered leaving and subsequently left. Age data were complete for all 268 participants. ① Independent‐samples *t* test; ② chi‐square test; ③ Mann–Whitney *U* test.

### 3.2. Exploratory Between‐Group Comparisons of Key Variable Scores

In exploratory comparisons, the MDS‐R total score and all four dimensions—individual responsibility, failure to act in the best interest of patients, value conflict, and harm to patients’ interests—were higher among nurses who had considered leaving (all *p* < 0.001).

The HECS total score and all five dimensions—nurse–nurse, nurse–patient, nurse–manager, nurse–hospital, and nurse–physician relationships—were lower among nurses who had considered leaving (all *p* < 0.001).

For moral resilience, nurses who had considered leaving had lower RMRS total scores and lower scores for personal integrity, moral adversity coping, moral efficacy, and relational integrity (all *p* < 0.05).

For social support, the SSRS total score, subjective support, and support utilization were lower among nurses who had considered leaving (all *p* < 0.05).

For moral courage, nurses who had considered leaving had lower scores for moral integrity, compassion and genuine presence, and moral responsibility (all *p* < 0.05). Detailed descriptive comparisons are presented in Table 2.

**TABLE 2:** Exploratory comparison of key variable scores by consideration of leaving (*n* = 268).

Variable	Total (*n* = 268)	Considered leaving (*n* = 104)	Never considered leaving (*n* = 164)	*t*/*Z*	*p* value
MDS‐R—total score	16.00 (1.00, 50.00)	32.50 (10.50, 71.50)	8.50 (0.00, 30.50)	5.791	< 0.001
Individual responsibility	2.00 (0.00, 11.75)	6.00 (1.00, 19.50)	0.00 (0.00, 5.75)	5.655	< 0.001
Failure to act in the best interest of patients	2.00 (0.00, 16.00)	7.50 (1.00, 19.75)	0.00 (0.00, 8.00)	5.344	< 0.001
Value conflict	6.00 (0.00, 18.00)	12.50 (4.00, 27.00)	2.00 (0.00, 14.00)	5.175	< 0.001
Harm to patients’ interests	0.00 (0.00, 4.00)	11.00 (3.00, 16.00)	0.00 (0.00, 1.00)	6.512	< 0.001
HECS—total score	109.00 (100.00, 122.00)	101.00 (99.00, 116.00)	115.00 (101.00, 124.00)	−4.648	< 0.001
Nurse–nurse	18.00 (16.00, 20.00)	17.00 (16.00, 19.00)	19.00 (16.00, 20.00)	−4.129	< 0.001
Nurse–patient	17.00 (16.00, 20.00)	16.00 (16.00, 18.00)	18.00 (16.00, 20.00)	−4.114	< 0.001
Nurse–manager	27.00 (24.00, 30.00)	25.00 (24.00, 28.00)	29.00 (24.00, 30.00)	−3.972	< 0.001
Nurse–hospital	26.00 (24.00, 30.00)	24.00 (23.00, 27.75)	28.00 (24.00, 30.00)	−4.830	< 0.001
Nurse–physician	21.00 (20.00, 25.00)	20.00 (19.00, 23.00)	22.00 (20.00, 25.00)	−4.261	< 0.001
RMRS—total score	48.46 ± 7.48	44.83 ± 6.89	50.77 ± 6.92	6.859	< 0.001
Personal integrity	8.85 ± 1.62	8.53 ± 1.46	9.05 ± 1.70	2.580	0.008
Moral adversity coping	12.43 ± 3.27	10.88 ± 3.10	13.41 ± 3.00	6.644	< 0.001
Moral efficacy	12.51 ± 3.07	11.95 ± 2.59	12.87 ± 3.29	2.528	0.012
Relational integrity	14.67 ± 3.64	13.46 ± 3.45	15.44 ± 3.56	4.484	< 0.001
SSRS—total score	41.75 ± 8.92	39.94 ± 8.52	42.89 ± 9.00	2.667	0.008
Subjective support	24.25 ± 5.24	23.41 ± 5.24	24.79 ± 5.18	2.105	0.036
Objective support	9.17 ± 3.58	8.63 ± 3.50	9.51 ± 3.60	1.968	0.050
Utilization	8.32 ± 1.99	7.89 ± 1.78	8.59 ± 2.07	2.928	0.004
NMCS—total score	75.91 ± 18.94	72.76 ± 16.55	77.90 ± 20.10	2.277	0.024
Moral integrity	25.44 ± 6.42	24.46 ± 5.67	26.07 ± 6.79	2.088	0.038
Compassion and genuine presence	18.41 ± 4.65	17.60 ± 4.11	18.93 ± 4.91	2.394	0.017
Moral responsibility	14.59 ± 3.82	13.95 ± 3.34	14.99 ± 4.06	2.274	0.024
Commitment to providing good care	17.47 ± 4.79	16.75 ± 4.30	17.92 ± 5.04	1.959	0.051

*Note:* The “considered leaving” group includes 102 nurses who considered leaving but did not leave and 2 who considered leaving and subsequently left.

Abbreviations: HECS, Hospital Ethical Climate Survey; MDS‐R, Moral Distress Scale–Revised; NMCS, Nurses’ Moral Courage Scale; RMRS, Rushton Moral Resilience Scale; SSRS, Social Support Rating Scale.

### 3.3. Predictive Modeling and Internal Validation

#### 3.3.1. Predictor Diagnostics and Penalized Logistic Model

Predictor redundancy was substantial: VIFs ranged from 1.45 to 11.75, with the highest value for the HECS nurse–hospital dimension. Spearman’s correlations showed several strong within‐scale relationships, particularly among HECS and NMCS dimensions. The strongest examples were HECS nurse–manager with nurse–hospital (*ρ* = 0.907), HECS nurse–nurse with nurse–patient (*ρ* = 0.886), and NMCS moral integrity with moral responsibility (*ρ* = 0.884). RMRS moral adversity coping and MDS‐R harm to patients’ interests were moderately inversely correlated (*ρ* = −0.421).

Across the 25 outer validation fits, RMRS moral adversity coping and MDS‐R harm to patients’ interests were selected in 100% of elastic‐net models. Their mean standardized coefficients were −0.417 and 0.364, respectively, and the direction was consistent in every fit. HECS nurse–hospital was selected in 92% of fits; SSRS support utilization and MDS‐R value conflict in 76% each; RMRS moral efficacy in 72%; and RMRS relational integrity in 64%. All other predictors were selected in 32% or fewer of fits (Table 3).

**TABLE 3:** Predictor diagnostics and elastic‐net selection stability across 25 outer validation fits.

Predictor	VIF	Mean standardized coefficient	Selected fits, *n* (%)
RMRS—moral adversity coping	1.94	−0.417	25 (100)
MDS‐R—harm to patients’ interests	4.63	0.364	25 (100)
HECS—nurse–hospital	11.75	−0.107	23 (92)
SSRS—support utilization	1.53	−0.071	19 (76)
MDS‐R—value conflict	4.27	0.074	19 (76)
RMRS—moral efficacy	1.71	−0.095	18 (72)
RMRS—relational integrity	1.76	−0.071	16 (64)
NMCS—compassion and genuine presence	8.36	−0.011	8 (32)
HECS—nurse–patient	7.16	−0.006	7 (28)
HECS—nurse–nurse	8.18	−0.016	6 (24)
SSRS—objective support	1.56	−0.009	6 (24)
MDS‐R—failure to act in patients’ best interests	5.95	−0.009	6 (24)
HECS—nurse–manager	7.18	−0.008	5 (20)
NMCS—moral responsibility	7.21	−0.005	5 (20)
NMCS—commitment to providing good care	8.13	−0.005	5 (20)
HECS—nurse–physician	5.31	−0.004	4 (16)
MDS‐R—individual responsibility	5.02	0.002	4 (16)
NMCS—moral integrity	8.48	0.001	3 (12)
SSRS—subjective support	1.71	0.002	3 (12)
RMRS—personal integrity	1.45	0.002	2 (8)

*Note:* Coefficients are averaged across all 25 outer fits; coefficients equal to zero after penalization are included in the mean.

Abbreviation: VIF, variance inflation factor.

#### 3.3.2. CART and Random Forest Stability

The CART root split was not invariant across the 25 outer validation fits. MDS‐R harm to patients’ interests was the root variable in 13 fits (52%; the split threshold was 1.5 in all 13 fits), whereas RMRS moral adversity coping was the root variable in 12 fits (48%; median threshold 12.5, interquartile range 12.5–13.5).

In test‐fold permutation analysis of the random forest, RMRS moral adversity coping produced the largest model‐specific mean decrease in AUC when permuted (0.037), followed by MDS‐R harm to patients’ interests (0.021). Both had positive importance in 96% of outer fits. The remaining predictors had smaller and less consistent importance values (Table 4).

**TABLE 4:** Random forest permutation importance across 25 outer validation fits.

Rank	Predictor	Mean AUC decrease	SD	Positive fits, *n* (%)
1	RMRS—moral adversity coping	0.037	0.019	24 (96)
2	MDS‐R—harm to patients’ interests	0.021	0.015	24 (96)
3	SSRS—objective support	0.006	0.006	20 (80)
4	NMCS—moral integrity	0.006	0.007	19 (76)
5	MDS‐R—failure to act in patients’ best interests	0.003	0.007	19 (76)
6	NMCS—moral responsibility	0.002	0.006	20 (80)
7	HECS—nurse–hospital	0.002	0.009	16 (64)
8	MDS‐R—individual responsibility	0.002	0.009	15 (60)
9	HECS—nurse–nurse	0.002	0.005	15 (60)
10	RMRS—moral efficacy	0.002	0.008	15 (60)
11	MDS‐R—value conflict	0.001	0.009	13 (52)
12	SSRS—support utilization	0.000	0.007	15 (60)
13	HECS—nurse–patient	0.000	0.005	16 (64)
14	RMRS—personal integrity	−0.001	0.004	10 (40)
15	NMCS—compassion and genuine presence	−0.001	0.005	12 (48)
16	HECS—nurse–manager	−0.001	0.007	13 (52)
17	NMCS—commitment to providing good care	−0.001	0.006	15 (60)
18	RMRS—relational integrity	−0.002	0.008	11 (44)
19	SSRS—subjective support	−0.003	0.005	3 (12)
20	HECS—nurse–physician	−0.003	0.004	5 (20)

*Note:* Importance is the mean decrease in test‐fold AUC after predictor permutation.

Because correlated predictors can share predictive information, permutation importance was interpreted as a model‐specific measure of predictive contribution.

#### 3.3.3. Comparative Performance and Calibration

Based on participant‐level averaged out‐of‐fold probabilities, random forest had the highest AUC (0.761, 95% CI 0.702–0.818), followed by penalized logistic regression (0.747, 95% CI 0.688–0.805) and CART (0.740, 95% CI 0.679–0.799). Paired bootstrap comparisons showed no statistically significant AUC differences: random forest versus penalized logistic regression, difference 0.013 (95% CI −0.021 to 0.047; *p* = 0.441); random forest versus CART, 0.021 (95% CI −0.009 to 0.048; *p* = 0.158); and penalized logistic regression versus CART, 0.007 (95% CI −0.028 to 0.044; *p* = 0.679).

Random forest also had the lowest Brier score (0.192, 95% CI 0.171–0.215), although its confidence interval overlapped those of penalized logistic regression (0.199, 95% CI 0.179–0.220) and CART (0.201, 95% CI 0.177–0.227). Calibration intercepts were near zero, indicating little systematic over‐ or underprediction on average. Calibration slopes were 1.198 for penalized logistic regression, 0.795 for CART, and 1.073 for random forest (Table 5; Figure 1); values closer to 1 indicate better agreement between the spread of predicted and observed risks. Calibration curves were close to the ideal line through much of the observed range but showed some overestimation at higher predicted probabilities, where observations were relatively sparse.

**TABLE 5:** Internally validated model performance based on averaged out‐of‐fold predictions.

Model	AUC (95% CI)	Average precision (95% CI)	Brier score (95% CI)	Calibration intercept (95% CI)	Calibration slope (95% CI)
Penalized logistic regression	0.747 (0.688–0.805)	0.638 (0.543–0.732)	0.199 (0.179–0.220)	0.071 (−0.252–0.433)	1.198 (0.859–1.628)
CART	0.740 (0.679–0.799)	0.625 (0.532–0.734)	0.201 (0.177–0.227)	−0.076 (−0.359–0.230)	0.795 (0.538–1.097)
Random forest	0.761 (0.702–0.818)	0.646 (0.555–0.751)	0.192 (0.171–0.215)	0.024 (−0.259–0.358)	1.073 (0.747–1.484)

*Note:* Metrics are calculated from participant‐level probabilities averaged across five outer repeats. Confidence intervals were obtained using 2000 subject‐level bootstrap resamples. AUC = area under the receiver operating characteristic curve; CART = classification and regression tree.

**FIGURE 1:**
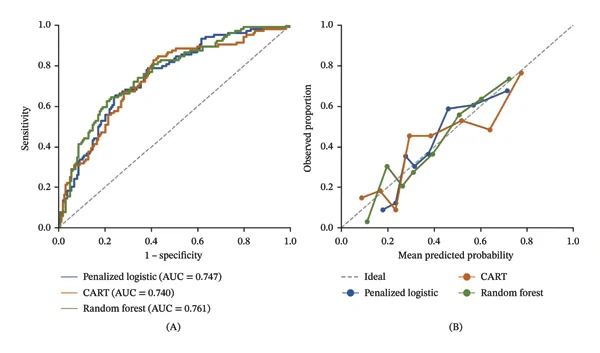
Receiver operating characteristic curves and calibration plots based on averaged out‐of‐fold predictions from repeated nested cross‐validation. (A) Discrimination. (B) Calibration.

#### 3.3.4. Threshold‐Dependent Classification Performance

Using thresholds selected within the corresponding outer training data, the mean accuracy, sensitivity, specificity, and F1 score across the five repeats were 0.690, 0.696, 0.685, and 0.635 for penalized logistic regression; 0.642, 0.629, 0.650, and 0.575 for CART; and 0.683, 0.656, 0.700, and 0.615 for random forest, respectively.

Taken together, the three models showed moderate discrimination and broadly comparable prediction error.

#### 3.3.5. Sensitivity Analyses

In the sensitivity analysis excluding the two participants who had considered leaving and subsequently left, 266 participants remained, including 102 with the positive outcome. MDS‐R harm to patients’ interests and RMRS moral adversity coping were selected in all 25 elastic‐net fits, with mean standardized coefficients of 0.436 and −0.448, respectively. CART root splits involved moral adversity coping in 13 of 25 fits and harm to patients’ interests in 12 of 25 fits. AUCs were 0.752 for penalized logistic regression and 0.736 for CART. In the extended elastic‐net model incorporating demographic and work‐related characteristics, harm to patients’ interests and moral adversity coping were selected in all 25 fits, with an internally validated AUC of 0.755. Demographic and work‐related variables showed little selection stability. Across both sensitivity analyses, the two ethics‐related signals remained the most stable predictors.

## 4. Discussion

This exploratory study of nurses working in infectious isolation wards identified two consistent dimension‐level signals associated with having considered leaving because of moral distress. Greater moral distress related to harm to patients’ interests and lower moral adversity coping were prominent across the models and remained so in sensitivity analyses. The findings draw attention both to nurses’ experiences of ethically difficult care and to their resources for responding to moral adversity. Because model discrimination was moderate, these associations are more useful for guiding further inquiry than for classifying individual nurses.

### 4.1. Moral Distress Related to Patients’ Interests

Previous studies examining moral distress at the overall level have linked it with nurses’ intentions to leave in different clinical contexts, including intensive care and the COVID‐19 pandemic [13, 34, 35], while also documenting its association with emotional exhaustion [15]. Our study extends this work by examining moral distress at the dimension level. Although all four moral distress dimensions differed between nurses who had and had not considered leaving, distress related to harm to patients’ interests provided the most consistent predictive information within this domain. By contrast, value conflict was selected relatively often in the penalized model but contributed little in random forest permutation analysis. These differences suggest that morally troubling experiences are not interchangeable when considering their predictive associations with thoughts of leaving.

This prominence may reflect the central place of patient advocacy and welfare in nursing practice. When nurses perceive that clinical decisions or work conditions compromise patients’ interests, they may experience tension between their professional responsibilities and what they feel able to do in practice. Our previous mixed‐methods study of oncology nurses also documented ethical tensions involving clinical directives, patients’ preferences, and nurses’ professional judgments [36]. Such tension could be relevant when nurses consider leaving because of moral distress. The harm‐related dimension captures perceived ethical difficulty, not verified patient harm. Examining specific sources of moral distress may therefore complement overall distress scores in identifying concerns that warrant managerial attention.

### 4.2. Moral Resilience and Moral Courage as Individual Ethical Resources

Previous studies have associated greater overall moral resilience with lower turnover intention and other adverse work outcomes [37, 38]. In our exploratory group comparisons, the resilience total score and all four dimensions were lower among nurses who had considered leaving. Their multivariable predictive contributions nevertheless differed. Moral adversity coping was consistently retained across elastic‐net fits and showed the largest random forest permutation importance, whereas the other resilience dimensions were less consistently informative. This contrast illustrates why an overall association with moral resilience should not be taken to imply equivalent contributions from each of its components.

The moral adversity coping dimension concerns how nurses respond to ethically challenging experiences, making it particularly relevant to a question about considering leaving because of moral distress. Its prominence identifies a promising focus for prospective research, rather than establishing a protective effect. Moral courage showed a different pattern. Although several dimensions differed between the groups, and prior research has linked moral courage with resignation‐related outcomes [39], they added little consistent predictive information alongside other ethical and contextual variables. Because moral courage and related constructs may overlap, lower model importance should not be interpreted as a lack of practical relevance.

### 4.3. Ethical Climate and Social Support as Contextual Resources

Prior research has associated more positive ethical climates [19, 20] and greater social support [21, 40, 41] with lower turnover intention. In our descriptive comparisons, all ethical climate dimensions and some social support measures differed between groups. In multivariable prediction, however, the nurse–hospital ethical climate dimension and support utilization were retained relatively often in elastic‐net models but contributed little to random forest discrimination. Strong correlations within the ethical climate scale suggest that these measures share substantial predictive information, making their individual rankings sensitive to the modeling approach.

This difference between group‐level association and incremental prediction does not diminish the practical importance of organizational conditions. Model importance describes what a variable adds in the presence of other correlated variables; it does not estimate the effect of changing workplace practices. The present findings therefore support continued attention to ethical climate and social support without singling out one contextual dimension as a consistently dominant predictor.

### 4.4. Implications for Nursing Management

For nursing management, these findings point to complementary areas for future evaluation. One is identifying and responding to care situations that nurses perceive as compromising patients’ interests. Interdisciplinary ethics case discussions, accessible channels for raising patient care concerns, and debriefings following difficult decisions could help managers understand these experiences and identify aspects of clinical practice that warrant review. Responding to concerns about care conditions matters because improving individual coping alone may not address the sources of ethical distress.

A second area for future evaluation is supporting nurses as they handle moral adversity, potentially through reflective supervision, peer consultation, and training in navigating ethical dilemmas. These approaches could be combined with responsive management and access to ethics consultation, rather than offered as stand‐alone individual solutions. Their feasibility and effects on subsequent consideration of leaving and retention need prospective evaluation. The model‐derived thresholds should not be used for clinical or staffing decisions.

## 5. Conclusions

Among nurses working in infectious isolation wards, moral distress related to harm to patients’ interests and lower moral adversity coping were the most internally stable predictors of consideration of leaving. Their stability in outcome and demographic/work‐related sensitivity analyses supports closer assessment of patient harm–related ethical distress and nurses’ capacity to cope with moral adversity in future retention research and nursing management. The models showed moderate discrimination, and the findings warrant prospective validation in larger external samples.

## 6. Limitations

This study has several limitations. Convenience sampling from selected hospitals in one Chinese province may limit generalizability, and external validation was not performed. The outcome was a single study‐specific self‐report item and did not capture the intensity, frequency, timing, or current immediacy of consideration of leaving; concurrent self‐report measurement may also introduce common method and recall or social desirability bias. The sample was modest relative to 20 correlated candidate predictors under contemporary prediction model development guidance [42], and the cross‐sectional design precludes causal inference. The very high internal consistency coefficients for the NMCS and HECS may also partly reflect item overlap.

## Funding

This work was supported by the Fujian Provincial Federation of Social Sciences (Grant Number FJ2025B066).

## Disclosure

The funding sources had no involvement in the design of the study, data collection, analysis, interpretation of results, or writing of the manuscript.

## Conflicts of Interest

The authors declare no conflicts of interest.

## Supporting Information

Additional supporting information can be found online in the Supporting Information section.

## Supporting information


**Supporting Information** The STROBE checklist is provided as supporting information.

## Data Availability

De‐identified data supporting the findings may be available from the corresponding author upon reasonable request, subject to applicable institutional and ethical requirements. Participant‐level data are not publicly available because the study consent and ethics arrangements did not provide for unrestricted public sharing. Analysis code is available from the corresponding author upon reasonable request.

## References

[1] Tamata A. T. and Mohammadnezhad M. , A Systematic Review Study on the Factors Affecting Shortage of Nursing Workforce in the Hospitals, Nursing Open. (2023) 10, no. 3, 1247–1257, 10.1002/nop2.1434. PMC9912424 36303066

[2] Lee J. E. , Kim S. R. , and Chun B. C. , Impact of the COVID-19 Pandemic on Infection Control Nurses: a Path Analysis of Job Stress, Burnout, and Turnover Intention, American Journal of Infection Control. (2024) 52, no. 6, 683–687, 10.1016/j.ajic.2024.01.001. 38218329

[3] Eweida R. S. , Khedr M. A. , Ghallab E. , Ibrahim N. , Khonji L. M. , and Ali E. A. , Effectiveness of Mental Health First Aid as a Supportive Tactic on Resilience and Organisational environment-related Factors Among Hospital Nurses in the Wake of COVID-19: the Search Continues, Journal of Research in Nursing. (2025) 30, no. 2, 168–184, 10.1177/17449871241275011. PMC11985474 40224355

[4] Gao X. , Jiang L. , Hu Y. , Li L. , and Hou L. , Nurses’ Experiences Regarding Shift Patterns in Isolation Wards During the COVID-19 Pandemic in China: a Qualitative Study, Journal of Clinical Nursing. (2020) 29, no. 21-22, 4270–4280, 10.1111/jocn.15464. PMC7460988 32810919

[5] Wynendaele H. , Clays E. , Peeters E. , DeJonghe Y. , Van Hecke A. , and Trybou J. , Understanding Turnover in Healthcare and Welfare Sectors of high-income Countries: an Umbrella Review, BMC Health Services Research. (2025) 25, no. 1, 10.1186/s12913-025-12966-5. PMC12139298 40474167

[6] Li Y. and Jones C. B. , A Literature Review of Nursing Turnover Costs, Journal of Nursing Management. (2013) 21, no. 3, 405–418, 10.1111/j.1365-2834.2012.01411.x. 23406301

[7] Hu H. , Wang C. , Lan Y. , and Wu X. , Nurses′ Turnover Intention, Hope and Career Identity: the Mediating Role of Job Satisfaction, BMC Nursing. (2022) 21, no. 1, 10.1186/s12912-022-00821-5. PMC8830989 35144604

[8] Liu Y. , Duan Y. , and Guo M. , Turnover Intention and Its Associated Factors Among Nurses: a multi-center cross-sectional Study, Frontiers in Public Health. (2023) 11, 10.3389/fpubh.2023.1141441. PMC10311009 37397760

[9] Zhang F. , Lin C. , Li X. et al., The Relationships Between Burnout, General Wellbeing, and Psychological Detachment with Turnover Intention in Chinese Nurses: a cross-sectional Study, Frontiers in Public Health. (2023) 11, 10.3389/fpubh.2023.1216810. PMC10399590 37546331

[10] Etti N. , Irit B. , and Michal I. , Caring for Patients with Infectious Diseases: Nurses’ Risk Perception, Moral Distress, Professional Ethos and Emotional Labor: a Mixed Methods Study, Journal of Clinical Nursing. (2025) 34, no. 8, 3133–3145, 10.1111/jocn.17750. 40119560

[11] Sanduni Nishara M. G. and Ariyasinghe Asurakkody T. , Ethical Challenges Experienced by Nurses During COVID-19: Are we Ready for Tomorrow?, BMC Nursing. (2025) 24, no. 1, 10.1186/s12912-025-03475-1. PMC12306059 40722179

[12] Corley M. C. , Elswick R. K. , Gorman M. , and Clor T. , Development and Evaluation of a Moral Distress Scale, Journal of Advanced Nursing. (2001) 33, no. 2, 250–256, 10.1111/j.1365-2648.2001.01658.x. 11168709

[13] Karakachian A. and Colbert A. , Nurses′ Moral Distress, Burnout, and Intentions to Leave: an Integrative Review, Journal of Forensic Nursing. (2019) 15, no. 3, 133–142, 10.1097/jfn.0000000000000249. 31436681

[14] Cerela-Boltunova O. , Millere I. , and Nagle E. , Moral Distress, Professional Burnout, and Potential Staff Turnover in Intensive Care Nursing Practice in Latvia—phase 1, International Journal of Environmental Research and Public Health. (2025) 22, no. 8, 10.3390/ijerph22081261. PMC12386003 40869846

[15] Orgambídez A. , Borrego Y. , Alcalde F. J. , and Durán A. , Moral Distress and Emotional Exhaustion in Healthcare Professionals: a Systematic Review and Meta-Analysis, Healthcare. (2025) 13, no. 4, 10.3390/healthcare13040393. PMC11855070 39997268

[16] Young P. D. and Rushton C. H. , A Concept Analysis of Moral Resilience, Nursing Outlook. (2017) 65, no. 5, 579–587, 10.1016/j.outlook.2017.03.009. 28434608

[17] Yi L. , Chen Z. , Jiménez-Herrera M. F. , Gan X. , Ren Y. , and Tian X. , The Impact of Moral Resilience on Nurse Turnover Intentions: the Mediating Role of Job Burnout in a cross-sectional Study, BMC Nursing. (2024) 23, no. 1, 10.1186/s12912-024-02357-2. PMC11437732 39334202

[18] Sadooghiasl A. , Parvizy S. , and Ebadi A. , Concept Analysis of Moral Courage in Nursing: a Hybrid Model, Nursing Ethics. (2018) 25, no. 1, 6–19, 10.1177/0969733016638146. 27098415

[19] Kim H. , Kim H. , and Oh Y. , Impact of Ethical Climate, Moral Distress, and Moral Sensitivity on Turnover Intention Among Haemodialysis Nurses: a cross-sectional Study, BMC Nursing. (2023) 22, no. 1, 10.1186/s12912-023-01212-0. PMC9969632 36842970

[20] Ammari N. and Gantare A. , Ethical Climate and Turnover Intention Among Nurses: a Scoping Review, Nursing Ethics. (2025) 32, no. 5, 1434–1457, 10.1177/09697330241296875. 39487744

[21] Liu Z. , Yan X. , Xie G. et al., The Effect of Nurses′ Perceived Social Support on Turnover Intention: the Chain Mediation of Occupational Coping self-efficacy and Depression, Frontiers in Public Health. (2025) 13, 10.3389/fpubh.2025.1527205. PMC11983550 40213429

[22] Xie M. , Ren X. , Zhao L. , Zhang M. , Jiang J. , and Dai Y. , The Influence of Nurses ′ Social Support on Ethical Dilemma, The Journal of Nursing Administration. (2014) 14, no. 11, 773–775.

[23] Breiman L. , Random Forests, Machine Learning. (2001) 45, no. 1, 5–32, 10.1023/A:1010933404324.

[24] Hanley J. A. and McNeil B. J. , The Meaning and Use of the Area Under a Receiver Operating Characteristic (ROC) Curve, Radiology. (1982) 143, no. 1, 29–36, 10.1148/radiology.143.1.7063747. 7063747

[25] Heinze K. E. , Hanson G. , Holtz H. , Swoboda S. M. , and Rushton C. H. , Measuring Health Care Interprofessionals′ Moral Resilience: Validation of the Rushton Moral Resilience Scale, Journal of Palliative Medicine. (2021) 24, no. 6, 865–872, 10.1089/jpm.2020.0328. 33196347

[26] Yang Q. , Zheng Z. , Pang S. et al., Translation of the Rushton Moral Resilience Scale and Its Validation in Healthcare Professionals, Journal of Nursing Science. (2022) 37, no. 15, 8–11.

[27] Hamric A. B. , Borchers C. T. , and Epstein E. G. , Development and Testing of an Instrument to Measure Moral Distress in Healthcare Professionals, AJOB Primary Research. (2012) 3, no. 2, 1–9, 10.1080/21507716.2011.652337.

[28] Sun X. , The Revision of the Chinese Version of the Nurse Ethical Dilemma Scale and Its Application among Nursing Staff, 2011, Shandong University.

[29] Numminen O. , Katajisto J. , and Leino-Kilpi H. , Development and Validation of Nurses′ Moral Courage Scale, Nursing Ethics. (2019) 26, no. 7-8, 2438–2455, 10.1177/0969733018791325. 30185132

[30] Wang S. , Wei L. , Zhang Y. et al., Translation and Psychometric Evaluation of the Nurse Moral Courage Scale in Chinese, Journal of Nursing. (2019) 34, no. 21, 92–95.

[31] Xiao S. , The Theoretical Basis and Research Application of the Social Support Rating Scale, Clinical Psychiatry Journal, 1994, no. 02, 98–100.

[32] Olson L. L. , Hospital Nurses′ Perceptions of the Ethical Climate of Their Work Setting, Image Journal of Nursing Scholarship. (1998) 30, no. 4, 345–349, 10.1111/j.1547-5069.1998.tb01331.x. 9866295

[33] Wang L. , The Chinese Translation and Application Study of the Hospital Ethical Climate Scale [doctoral dissertation], 2018, Zhengzhou University.

[34] Sheppard K. N. , Runk B. G. , Maduro R. S. et al., Nursing Moral Distress and Intent to Leave Employment During the COVID-19 Pandemic, Journal of Nursing Care Quality. (2022) 37, no. 1, 28–34, 10.1097/ncq.0000000000000596. PMC8607915 34538814

[35] Ahmad A. M. , Bani-Issa W. , Refaat F. , Al-Tamimi M. S. , and Al-Yafeai T. M. , Moral Distress and Intention to Leave Among Intensive Care Unit Nurses in the United Arab Emirates, International Journal of Nursing Sciences. (2025) 12, no. 6, 581–587, 10.1016/j.ijnss.2025.08.003. PMC12684761 41367589

[36] Zhan S. , Yu N. , Wang J. et al., How Moral Resilience Shapes Oncology Nurses’ Responses to Ethical Challenges: a Mixed-Methods Study, Cancer Nursing. (2026) 10.1097/NCC.0000000000001602. 42405588

[37] Galanis P. , Moisoglou I. , Katsiroumpa A. et al., Moral Resilience Reduces Levels of Quiet Quitting, Job Burnout, and Turnover Intention Among Nurses: Evidence in the Post COVID-19 Era, Nursing Reports. (2024) 14, no. 1, 254–266, 10.3390/nursrep14010020. PMC10885038 38391065

[38] Kovanci M. S. and Atli Özbaş A. , Moral Resilience and Intention to Leave: Mediating Effect of Moral Distress, Nursing Ethics. (2025) 32, no. 3, 864–874, 10.1177/09697330241272882. 39133181

[39] Li H. , Guo J. , Ren Z. et al., Moral Courage Level of Nurses: a Systematic Review and meta-analysis, BMC Nursing. (2024) 23, no. 1, 10.1186/s12912-024-02082-w. PMC11295526 39090605

[40] Chen Y. , Zhou X. , Bai X. et al., A Systematic Review and meta-analysis of the Effectiveness of Social Support on Turnover Intention in Clinical Nurses, Frontiers in Public Health. (2024) 12, 10.3389/fpubh.2024.1393024. PMC11187297 38903567

[41] Kim H. and Kim E. G. , A meta-analysis on Predictors of Turnover Intention of Hospital Nurses in South Korea (2000–2020), Nursing Open. (2021) 8, no. 5, 2406–2418, 10.1002/nop2.872. PMC8363357 33826252

[42] Riley R. D. , Snell K. I. E. , Ensor J. et al., Minimum Sample Size for Developing a Multivariable Prediction Model: Part II—Binary and time-to-event Outcomes, Statistics in Medicine. (2019) 38, no. 7, 1276–1296, 10.1002/sim.7992. PMC6519266 30357870

